# Estimating cost of in-patient medical care for stroke using Casemix data

**DOI:** 10.1186/1472-6963-12-S1-P10

**Published:** 2012-11-21

**Authors:** Nor Azlin Mohd Nordin, Amrizal Muhamad Nur, Saperi Sulong, Syed Aljunid

**Affiliations:** 1 United Nations University International Institute for Global Health, Kuala Lumpur, Malaysia; 2 International Casemix & Clinical Coding Centre, Universiti Kebangsaan Malaysia, Kuala Lumpur, Malaysia

## Background

There is a lack of data on financial impact of stroke despite the increase in hospital admissions due to this disease in Malaysia. This study aimed to estimate the cost of medical care for patients hospitalised in a main teaching hospital following a stroke.

## Method

A retrospective analysis was conducted using data of stroke patients maintained by the Casemix Unit of Universiti Kebangsaan Malaysia Medical Centre. Variables studied were the patients’ demography, clinical profiles and length of hospital stay. Cost evaluation was carried out from the hospital perspective using Top-down costing approach. Data was analysed with the use of SPSS version 19.

## Results

Data of 748 stroke patients were retrieved for analysis. The average length of hospital stay was 6.4 (SD: 3.1) days. The mean cost of medical care was RM 3696.40 (SD: 1842.10) per stroke patient per admission. Human resources made up the highest cost component (RM 1343.90, SD: 669.8) or 36% of the total cost of care, followed by medications (RM 867.30, SD: 432.40) and laboratory tests (RM 337.90, SD: 168.40). The cost increased by 15% when patient suffered from moderately severe stroke and further increased by 52% in the highest level of severity compared to mild stroke (p < 0.05). No significant differences were found when comparing costs between the sub-groups of age, gender and stroke sub-types.

## Conclusion

There is a substantial cost involved in the care of in-patient stroke in this study. Further research involving other health care settings are required to better estimate the financial impact of stroke to this country.

